# Diagnostic yield of intraoperative frozen sections obtained through robot-assisted stereotactic biopsy of brain lesions

**DOI:** 10.3389/fneur.2025.1544613

**Published:** 2025-06-10

**Authors:** Zhijie Chen, Wangchi Luo, Yu Lin, Bin Deng, Xubiao Zhang, Yongqin Zeng, Tao Lin, Da Liu

**Affiliations:** ^1^Department of Neurosurgery, Guangdong Sanjiu Brain Hospital, Guangzhou, China; ^2^Department of Neurology, The Fourth Affiliated Hospital of Guangzhou Medical University, Guangzhou, China; ^3^Department of Laboratory, Guangdong Sanjiu Brain Hospital, Guangzhou, China

**Keywords:** brain tumor, stereotactic biopsy, robotics, frozen section, diagnostic yield

## Abstract

**Objective:**

To evaluate the diagnostic yield and diagnostic accuracy of intraoperative frozen sections obtained through robot-assisted stereotactic biopsy of brain lesions.

**Methods:**

The medical records of 87 patients who underwent 89 robot-assisted stereotactic biopsies of brain lesions at our institution between June 2015 and January 2024 were retrospectively reviewed. All patients were assessed using hematoxylin/eosin (HE) staining of intraoperative frozen sections, and intraoperative immunohistochemical examination when necessary. A final diagnosis derived from integrated diagnostics (neoplastic diseases) or final histopathologic examination (non-neoplastic diseases) was the ‘gold standard’. Intraoperative frozen section results were divided into 3 categories: confirmed diagnosis, tentative diagnosis, and misdiagnosis. Subgroup analyses of negative intraoperative frozen section results, tentative diagnoses or misdiagnoses were conducted stratified by lesion size and lesion type.

**Results:**

Mean turn-around time for intraoperative frozen sections was 26 ± 5.6 min (range, 20–62 min). 1 (FS-1) to 4 (FS-N) (median, 1) intraoperative frozen sections were evaluated per patient. There was a significant increase in positive results from FS-1 (79.77%; *n* = 71/89) to FS-N (92.13%; *n* = 82/89) (*p* = 0.018). FS-1 results were negative in 18 (20.22%) patients. Among these, FS-N results were positive after adjusting the puncture depth or changing the target in 11 patients. The overall concordance rate of intraoperative frozen section to final diagnosis was 91.1% (confirmed diagnosis, *n* = 73; tentative diagnosis, *n* = 8). Intraoperative immunohistochemistry was performed on the frozen sections of 38 patients (42.7%). Among the patients with negative FS-1 results, tentative diagnoses or misdiagnoses, there were 12, 6 and 7 patients with medium sized lesions, respectively. Eight patients with negative FS-1 results had high-grade glioma.

**Conclusion:**

The diagnostic yield of intraoperative frozen sections obtained through robot-assisted stereotactic biopsy of brain lesions is high. If the first frozen section result is negative, additional specimens should be obtained after adjusting the puncture depth or the target. Lesions that are difficult to distinguish morphologically on HE staining may be examined using intraoperative immunohistochemistry. High-grade glioma may be more prone to tentative or misdiagnosis due to heterogeneity of the lesion.

## 1 Introduction

The World Health Organization (WHO) has identified more than 100 types of benign and malignant brain and central nervous system (CNS) lesions that are unique and histologically complex (1). These lesions comprise an estimated 1.9% of all cancers and cause 2.5% of all cancer deaths (2). Brain and CNS lesions are associated with high morbidity and mortality, necessitating accurate diagnosis and timely treatment.

Accurate diagnosis of brain lesions has been facilitated by technological advancements, but the misdiagnosis rate remains high at approximately 30% (3). Stereotactic brain biopsy is a frequently performed, highly efficient, minimally invasive brain surgery that enables precise sampling of pathological brain tissue for histological analysis using a stereotactic needle (4–8). The robotized technique allows the programming of accurate and predefined trajectories, which can be repeated with the same precision, or reprogrammed, minimizing errors that may be made by surgeons (7). Evidence suggests that robot-guided stereotactic techniques are safe and effective for biopsies of brain lesions; however, there is a risk of misdiagnosis or false negative diagnosis due to off-target sampling during the needle puncture procedure.

Intraoperative frozen-section histology can be used to determine the presence of pathological tissue in biopsy specimens (7). Diagnostic yield of stereotactic brain biopsy varies from 72.8 to 100%, but is consistently over 86.7% using intraoperative frozen sections (5, 9–13). Intraoperative frozen section diagnosis is cost-effective and can provide reliable information (14); however, some studies suggest that it can be time-consuming, provides insufficient specimens for a definitive histological interpretation, can increase complications, and requires a well-trained pathologist (15).

Surgeons must strive for the highest diagnostic yield and diagnostic accuracy when treating brain lesions, while minimizing the morbidity and mortality that can be associated with intracranial biopsy (14). To further inform surgeons involved in stereotactic biopsy procedures, this study describes our experience evaluating intraoperative frozen sections obtained through robot-assisted stereotactic biopsy of brain lesions.

## 2 Methods

### 2.1 Data source

The medical records of patients who underwent robot-assisted stereotactic biopsy of brain lesions at our institution between June 2015 and January 2024 were retrospectively reviewed. Preoperatively, patients’ physical fitness was assessed using 2016 American Society of Anesthesiologists (ASA) classification criteria, and all patients underwent a preoperative head computed tomography (CT) scan and magnetic resonance imaging (MRI) (T1-weighted, T2-weighted, T2-weighted fluid attenuated inversion recovery [FLAIR], contrast-enhanced), with some patients also undergoing positron emission tomography (PET)-CT, MR spectroscopy (MRS), arterial spin labeling (ASL), and if necessary, lumbar puncture for examination of cerebrospinal fluid.

Inclusion criteria were: (1) brain lesion that was difficult to diagnose with non-invasive imaging; (2) lesion located next to sensitive structures, such that open surgery was associated with an extremely high risk of loss of function; (3) diagnosis on imaging required pathological confirmation before radiotherapy or chemotherapy (e.g., germ cell tumor, lymphoma); (4) highly malignant lesion or postoperative recurrence of a benign tumor with worsening symptoms, with family refusing repeat craniotomy; (5) need to differentiate between tumor progression and radiation necrosis after comprehensive treatment of an intracranial tumor; and (6) patient and family provided consent for robot-assisted stereotactic biopsy. Exclusion criteria were: (1) ASA class ≥ V; (2) contraindication to surgery due to coagulation dysfunction; (3) imaging suggestive of brain herniation; or (4) patient and family refused robot-assisted stereotactic biopsy.

### 2.2 Surgical procedure and frozen section

Preoperatively, multidisciplinary discussions involved the Departments of Neurosurgery, Neurology, Radiology, Oncology, and Pathology. Targeted biopsies were performed along trajectories that avoided functional areas of the brain. The biopsy procedures were assisted by the SINO robot (Sinovation Medical, China). After biopsy, normal saline solution was dripped from the end of the puncture needle to promote hemostasis.

In this study, robot-assisted stereotactic biopsies of brain lesions were performed by a team of experienced neurosurgeons from our hospital’s Department of Neurosurgery. A total of four neurosurgeons participated in the 89 robot-assisted stereotactic biopsy procedures. All these surgeons had undergone specialized training to ensure the precision and consistency of the surgical procedures.

The nature and color of stereotactic puncture specimens were observed. 1–2 samples (0.1 cm × 1 cm) with an abnormal appearance (e.g., gelatinous) were sent for intraoperative frozen section examination, and 2–4 samples were retained for final histopathologic examination and molecular testing. For neoplastic diseases, if the final histopathologic examination could not make a definite diagnosis, supplementary genetic testing using fluorescence *in situ* hybridization (FISH), gene rearrangement, and polymerase chain reaction (PCR) was performed.

Intraoperative frozen sections were cut sequentially at 0.2 cm intervals using a LEICA CM1950 cryostat (Leica, Germany), with the temperature set at −19 to −20°C, and a slice thickness of 4 μm. The first intraoperative frozen section was designated FS-1, and subsequent intraoperative frozen sections were designated FS-N (*N* = 2, 3 or 4). 2–3 slices per tissue were examined under a light microscope after hematoxylin/eosin (HE) staining to observe specimen morphology and make a preliminary diagnosis. If intraoperative diagnosis was difficult on an HE stained frozen section, immunohistochemistry was performed. Remaining slices were paraffin embedded for final histopathologic examination. This ensured consistency between frozen section analysis and final permanent pathology.

### 2.3 Definitions

FS-1 represents the first intraoperative frozen section, while FS-N represents subsequent intraoperative frozen sections, where *N* is 2, 3, or 4, depending on the total number of frozen sections evaluated for each patient. At least one frozen section (FS-1) was evaluated for each patient, with a maximum of four frozen sections (FS-N, *N* = 2, 3, or 4) evaluated. If the result of FS-1 was negative, the puncture depth was adjusted or the target point was changed according to the location and characteristics of the brain lesion to obtain additional sections (FS-2 to FS-4). This process was based on the professional judgment of the surgeon according to the characteristics of the brain lesion and results from the preliminary section.

### 2.4 Diagnostic standard

Intraoperative frozen sections were evaluated against integrated diagnoses (incorporating molecular parameters) as the gold standard. Neoplastic lesions were classified according to the 2016 WHO Classification of CNS Tumors (16), with consideration of the 2021 WHO Classification updates (17) where relevant.

Intraoperative frozen section results were divided into 3 categories, as previously described (18, 19):

(1) Confirmed diagnosis: intraoperative diagnosis matched the integrated final diagnosis in both tumor identification and grading. For gliomas, this included correct identification of molecular features when available (IDH status, 1p/19q codeletion), recognizing the 2021 WHO Classification’s simplified categories for IDH-mutant gliomas and refined molecular criteria for IDH-wildtype glioblastoma. For lymphomas, this required accurate identification of lymphoid proliferation with supporting morphological and immunohistochemical evidence.
(2) Tentative diagnosis: correct identification of lesion nature but incomplete classification or grading. This included cases where definitive diagnosis required additional molecular testing unavailable intraoperatively, particularly relevant as the 2021 WHO Classification emphasizes molecular parameters for definitive diagnosis.
(3) Misdiagnosis: false positive/negative results or incorrect tumor identification.

Both confirmed and tentative diagnoses were considered correct. Multiple biopsies with identical results were combined, and when both high-grade and low-grade components were present, the highest grade was reported.

### 2.5 Statistical analyses

Analyses were performed with SPSS v26.0 using descriptive statistics. Data are reported as means (± standard deviations) or ranges for quantitative variables, and as absolute numbers and/or percentages for categorical variables.

## 3 Results

### 3.1 Patients

This study included 87 patients (56 males and 31 females), with a mean age of 39.1 ± 21.6 years (range, 3.2–76 years). 73 patients were ASA class I-II, 12 patients were ASA class III, and 2 patients were ASA class IV. All patients had brain lesions, 56 patients had multiple or diffuse lesions (involving multiple brain lobes) and 31 patients had single lesions. Mean diameter of the lesions was 5.12 ± 2.90 cm (range, 0.8 ~ 12.5 cm). Two patients had negative biopsy results and underwent a second robot-assisted stereotactic biopsy; therefore, there were a total of 89 biopsy procedures of 97 target lesions. Lesions were categorized into three types in accordance with their longest diameters: small (< 2 cm), medium (2–5 cm), and giant (> 5 cm). Patients’ demographic and clinical characteristics and characteristics of the lesions are summarized in Table 1.

**Table 1:** Patients’ demographic and clinical characteristics and characteristics of the lesions.

Characteristic (*N* = 87)	Value	Characteristic (*N* = 89)	Value
Age (years)		Biopsy region	
Mean ± SD	39.1 ± 21.6	Frontal lobe	22
Range	3.2–76	Temporal lobe	9
Gender		Parietal lobe	2
Female	40	Occipital lobe	3
Lesion size (cm)	5.12 + 2.99 cm (1–12 cm)	Thalamus and/or basal ganglia	28
Lesion size		Midbrain	7
Small (≤2 cm)	6	Pons	13
Medium (2–5 cm)	45	Medulla oblongata	0
Giant (≥5 cm)	36	Cerebellum	2
Sides involved		Corpus callosum	3
Right	24	Other regions*	2
Left	26	Biopsy side	
Bilateral	37	Left	30
Lesion presentation		Right	54
Multiple or diffuse lesions	56	Bilateral	5
Single	31		
Brain regions involved			
Limited to 1 region	16		
2–3 regions	31		
> 3 regions	30		

*Other regions include optic chiasm, optic tract, fourth ventricle, septum pellucidum, lateral ventricles, etc.

### 3.2 Diagnostic yield of the stereotactic biopsy procedure

**Figure 1:**
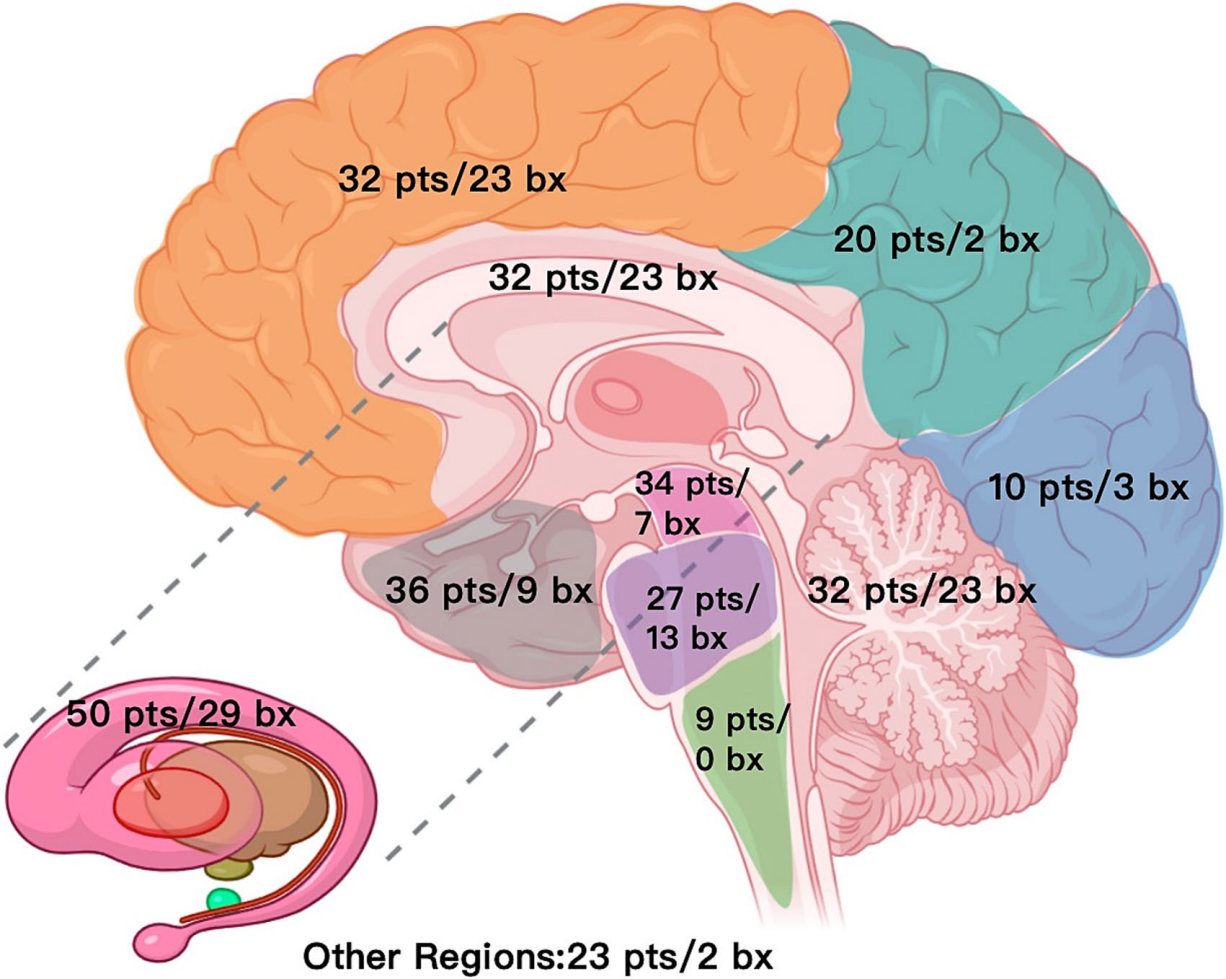
Distribution map of lesions and biopsy targets across brain regions (*N* = 89). pts./bx, patients involved/biopsies, Other regions include optic chiasm, optic tract, fourth ventricle, septum pellucidum, lateral ventricles, etc.

**Figure 2:**
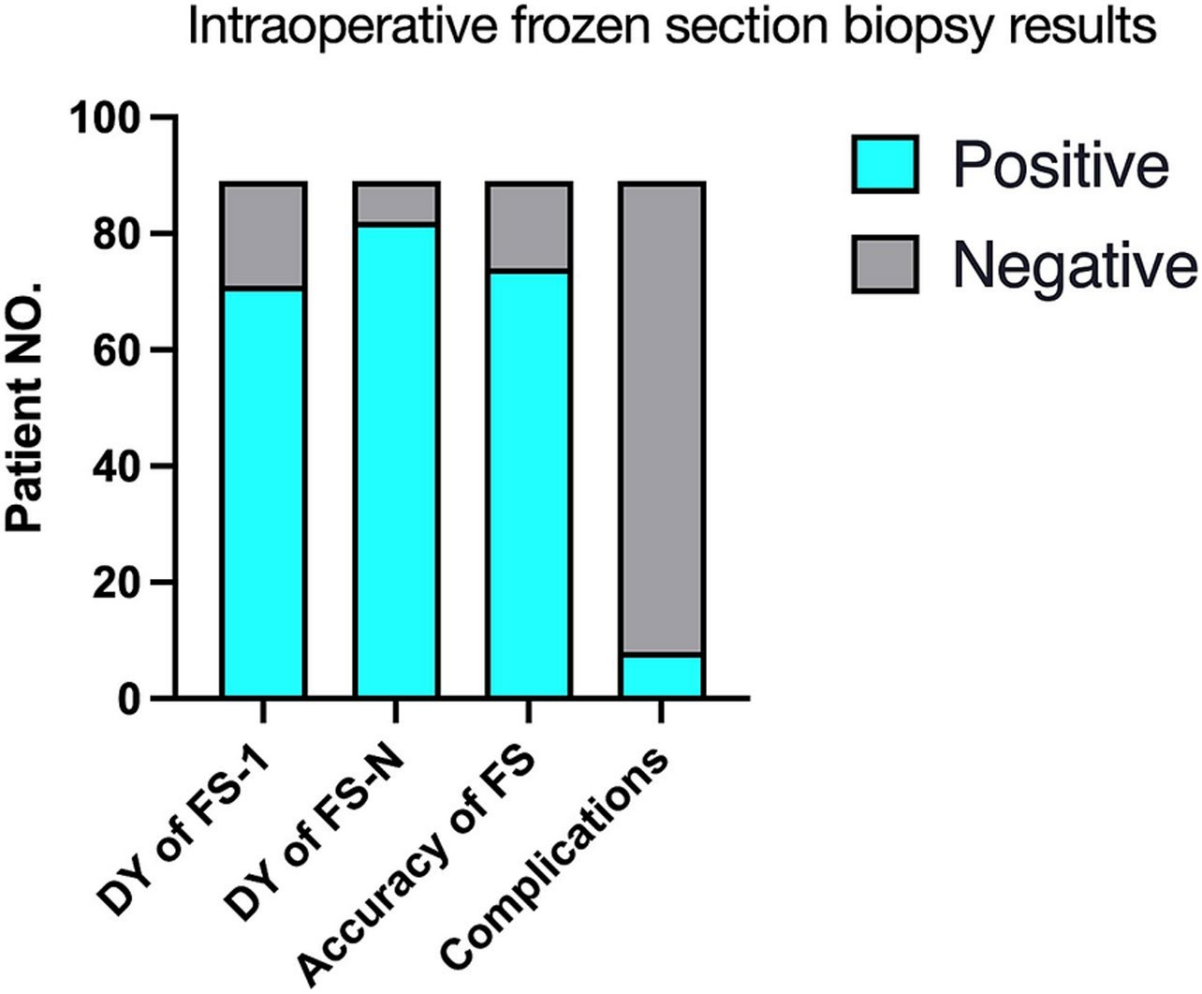
Intraoperative frozen section biopsy results (*N* = 89). FS-1, the first frozen section biopsy; FS-N, 1–4 frozen section biopsies; FS, frozen section; DY, Diagnostic yield.

Intraoperative frozen section biopsy results are summarized in Table 2, Figures 1 and 2. Mean turn-around time for intraoperative frozen sections was 26 ± 5.6 min (range, 20–62 min). 1 to 4 (median, 1) intraoperative frozen sections were evaluated per patient.

**Table 2:** Intraoperative frozen section biopsy results (*N* = 89).

Variable	Case no.	Percentage	*p*-value
DY of first FS	71	79.77%	*P* = 0.018*
DY of FS	82	92.13%	
DY of final diagnosis	85	96.55%	
Accuracy of FS (*N* = 89)	73	82.02%	
Concordance of FS (*N* = 89)	81	91.01%	*p* = 0.29#
Concordance of radiology (*n* = 87)	69	79.31%	
IHC applied in FS	38	42.69%	/
Diagnosis results			
Confirmed diagnosis	74	83.14%	/
Tentative diagnosis	7	7.87%	/
Misdiagnosis	8	8.99%	/
False negative FS	6	6.7%	/
False positive FS	2	2.2%	/
Diagnosis (*n* = 87)			
Grade I-II glioma	9	10.3%	/
Grade III-IV glioma	43	49.42%	/
Lymphoma	26	29.89%	/
Germ cell tumor	4	4.6%	/
Inflammation	3	3.45%	/
Brain tissue	1	1.15%	/
Gliosis	1	1.15%	/

*Comparison of first frozen section diagnostic yield and final frozen section diagnostic yield.

#Comparison of frozen section diagnostic concordance rate and radiology diagnostic concordance rate.

DY, diagnostic yield; IHC, immunohistochemistry; FP, frozen section.

The overall concordance rate of intraoperative frozen section to final diagnosis was 91.1% (confirmed diagnosis, *n* = 73; tentative diagnosis, *n* = 8). 8 patients were misdiagnosed on intraoperative frozen section, including 5 cases with negative intraoperative frozen section results. Intraoperative immunohistochemistry was performed on the frozen sections of 38 patients (42.7%) to clarify the nature of their lesions.

There was a significant increase in positive results from FS-1 (79.77%; *n* = 71/89) to FS-N (92.13%; *n* = 82/89) (*p* = 0.018, Table 2). FS-1 results were negative in 18 (20.22%) patients. Among these, FS-N results were positive after adjusting the puncture depth or changing the target in 11 patients or had the same negative interpretation in the other 7 patients.

Six patients had postoperative hematoma in the surgical area, including 5 patients with asymptomatic bleeding. One patient had severe delayed cerebral hemorrhage, which resulted in death. One patient with diffuse glioma experienced a postoperative seizure, which led to a sharp rise in intracranial pressure along with diffuse brain swelling, cerebral herniation, and death. Details are shown in Table 3. No other patients had surgery-related neurological dysfunction or intracranial infection.

**Table 3:** Complications of robot-assisted stereotactic biopsy.

Variable	Case no.	Percentage
Complications (*n* = 89)		
Small bleed	6	6.74%
Symptomatic bleeding	2	2.22%
Inflammation	1	1.11%
Pneumonia	2	2.22%
Dead	2	2.22%
Epilepsy	1	1.11%

Subgroup analyses of negative FS-1 results, tentative diagnoses or misdiagnoses were conducted stratified by lesion size and lesion type. 18 patients had negative FS-1 results, including 12 patients with medium sized lesions, 5 patients with giant lesions, 8 patients with high-grade glioma, 5 patients with low-grade glioma and 3 patients with lymphoma. Of these, 3 patients each with medium and giant lesions, and 4 patients with high-grade glioma had the same negative interpretation on FS-N results. All the patients with lymphoma had positive FS-N results. Eight patients had a tentative diagnosis, including 6 patients with medium sized lesions, 2 patients with giant lesions, and 5 patients with high-grade glioma. Eight patients were misdiagnosed, including 7 patients with medium sized lesions, 1 patient with a giant lesion, and 5 patients with high-grade glioma (Table 4).

**Table 4:** Subgroup analyses of intraoperative FS-1 results reported as negative, tentative diagnoses or misdiagnoses.

Variable	Negative on FS-1 (*N* = 18)	Negative on FS-N (*N* = 7)	Tentative diagnosis (*N* = 8)	Misdiagnosis (*N* = 8)
Lesion size				
Small (≤2 cm)	1	1	0	0
Medium (2–5 cm)	12	3	6	7
Giant (≥5 cm)	5	3	2	1
Lesion types				
Grade I–II glioma	5	1	2	0
Grade III–IV glioma	8	4	5	5
Lymphoma	3	0	1	2
Germ cell tumor	1	1	0	1
Inflammation	0	0	0	0
Brain tissue	1	1	0	0
Gliosis	0	0	0	0

### 3.3 Pathological diagnosis

Among the 87 patients, final diagnoses included glioma (*n* = 52; WHO grade I-II, *n* = 9; WHO grade III-IV, *n* = 43), lymphoma (*n* = 26), germ cell tumor (*n* = 4), inflammation (*n* = 3), gliosis (*n* = 1), and normal brain tissue (*n* = 1). For one patient, FS-1-4 results were reported as negative, a qualitative diagnosis was difficult on routine pathology, and the patient was ultimately diagnosed with T-cell lymphoma through genetic testing. One patient with recurrent low-grade glioma underwent biopsy of two targets, and the results were high-grade glioma and low-grade glioma. The patient was diagnosed as high-grade glioma.

Ten patients with lymphoma had received steroid treatment before the biopsy procedure (range, 2 weeks - 6 months). Among these, one patient was diagnosed with inflammation on intraoperative frozen section, but the final diagnosis was lymphoma. One patient was negative on FS-1 and positive on FS-N, while the other patients were all positive on FS-1.

## 4 Discussion

This study evaluated the diagnostic yield and diagnostic accuracy of intraoperative frozen sections obtained through robot-assisted stereotactic biopsy of brain lesions. Findings showed mean turn-around time for intraoperative frozen sections was 26 ± 5.6 min (range, 20–62 min). The diagnostic yield of intraoperative frozen sections was 91.01%. In accordance with previous studies (6, 20, 21), the diagnostic yield was increased by examining multiple tissue specimens, after the target or puncture depth had been adjusted (79.77% for FS-1 increasing to 92.13% for FS-N) (6, 20, 21). The diagnostic yield of preoperative imaging (MR and/or CT) was 79.31% (*n* = 69/87), with potential for misdiagnosis if treatment decision-making had been based on preoperative imaging alone. FS-1 results were negative in 18 (20.22%) patients; of these, FS-2 results were positive in 11 patients. This may be because many lesions were located at functional areas (e.g., brainstem, thalamus, basal ganglia) and the characteristics of FS-1 were only representative of the lesion periphery. The remaining 7 patients were still negative on FS-3 or FS-4. Some of these negative results were false negatives, as the diagnostic yield of the ‘gold standard’ approach of integrated diagnostics (neoplastic diseases) or histopathological examination (non-neoplastic diseases) was 96.63% (*n* = 86/89). Lesions that were difficult to distinguish morphologically on HE staining were examined immediately using intraoperative immunohistochemistry, adding to the turn-around time for intraoperative frozen sections. The 38 patients that underwent intraoperative immunohistochemistry included 21 of 26 (80.77%) patients with lymphoma, 13 patients with high-grade glioma, 3 patients with low-grade glioma, and 1 patient with germ cell tumor. Subgroup analyses showed that most patients with negative FS-1 results, tentative diagnosis or misdiagnosis, had medium sized lesions, and that high-grade glioma was prone to tentative and misdiagnosis, possibly due to the heterogeneity of the lesion (22, 23). These results imply that intraoperative frozen section obtained through robot-assisted stereotactic biopsy has clinical utility for determining the nature of brain lesions.

Currently, intraoperative frozen sections are not routinely obtained through stereotactic brain biopsy. Factors limiting widespread adoption of this approach include the small amount of biopsy tissue, prolonged operative-time, the relatively high technical requirements for frozen sections, the potential for false negative or false positive diagnoses due to errors in sampling and processing and varied experience of pathologists, and the high incidence of complications such as cerebral hemorrhage and dysfunction (15, 24–26). A search of the literature published in English since 1995 on the PubMed Medline database using the keyword “stereotactic brain biopsy,” showed only 11 studies reported on the use of intraoperative frozen sections (5, 9, 10, 12, 14, 21, 27–30) (Table 5). In these studies, the diagnostic yield ranged from 70.1–98.2%, and the diagnostic yield of final pathology was 87.6–98.7% (5, 9, 10, 12, 14, 21, 27–30). One study of MRI-based robot-assisted stereotactic biopsy in which the majority of patients had an intraoperative pathologic diagnosis with frozen sections showed that complication rates did not increase with the number of biopsy sites and complications were not associated with the number of biopsy samples (5).

**Table 5:** Studies that have applied frozen sections in stereotactic biopsy of brain lesions in the past 30 years.

No.	Reference	FS applied	DY of FS	DY of final pathology	Concordance rate of FS	Surgical technique	Surgical equipment	Number of biopsies
1	Current study	All cases	96.65%	96.65%	91.3%	Robot	ROSA, SINO	89
2	Hayden et al. (12)	All cases	78%	86.7%	75%	Frame	BRW, CRW	75
3	Brainard et al. (21)	All cases	84.04%	96.27%	88.83%	Frame	BRW, CRW, COMPASS	188
4	Kim et al. (28)	92%	90.1%	91.7%	79%	Frame	Riechert-Mundinger	308
5	Gralla et al. (27)	All cases	98.2%	94.8%	96.5%	Frameless	Stealth Treon	57
6	Woodworth et al. (9)	All cases	/	91%	70%	Frame	Leksell	160
			/	89%	78%	Frameless	FreeGuide	110
7	Dammers et al. (14)	8.9%	/	98.2%	/	Frameless	Stealth Treon	164
8	Mader et al. (30)	51.3%	75.4%	92.4%	78.7%	Frame	Leksell	110
9	Taweesomboonyat et al. (10)	76%	70.1%	87.6%	81%	Frameless	VarioGuide	85
10	Legnani et al. (29)	Some cases	/	97.4%	/	Micro-robot	iSYS1	39
11	Shofty et al. (32)	90.9%	/	96.8%	/	Frameless	VarioGuide	376
12	Zanello et al. (16)	Most cases	97.2%	98.7%	/	Robot	Neuromate	324
13	Majovsky et al. (36)	37.6%	66.67%	66.67%	59.09%	Frame, Frameless	CRW, VarioGuide	125

Several factors can lead to false negatives or misdiagnosis on intraoperative frozen sections. In the current study, false negatives or misdiagnosis occurred as (1) the scalp incision and trephination through which the biopsy needle was advanced did not align with the lesion; (2) the surgeon misinterpreted preoperative imaging and selected an atypical area as the target site; (3) the surgeon chose a target that was representative of the lesion periphery to avoid functional areas; and/or (4) improper robot operation. In our early cases, we overlooked a critical technical detail regarding the Sedan side-cutting biopsy needle. This needle (198 mm working length) has a 2 mm blind tip and a 10 mm side-cutting window. To align the window’s center with the target, we should have set the robotic working distance to 190–192 mm rather than the full 198 mm. This miscalculation likely contributed to some false-negative results in our initial series of biopsies.

Misinterpretation of intraoperative frozen sections can also impact the accuracy of diagnosis (31). To avoid false negatives or misdiagnosis, we recommend: (1) retrieving a specimen from the center of a small or medium sized lesion, but avoiding the central necrotic area for large glioblastoma (32, 33); (2) ensuring the specimen is representative of a brain lesion, with a fish-like appearance in contrast to the milky white appearance of healthy brain tissue; (3) obtaining several specimens by rotating the needle in different directions or varying the depth slightly; and (4) diagnosing intraoperative frozen sections jointly by multiple experienced pathologists.

Intraoperative cytological methods (cytology smear and touch imprint) are also used in stereotactic biopsy to assist in determining the nature of CNS lesions (12). Applying both cytological methods and frozen sections may improve intraoperative diagnostic accuracy. In one study, touch imprint, cytology smear, and frozen sections had a diagnostic accuracy of 78.4, 89.2, and 75.7%, respectively, and an overall diagnostic accuracy of 96% for CNS lesions (34). In another study, frozen sections and cytology smear had a diagnostic accuracy of 84.6 and 76.9%, respectively and an overall diagnostic yield of 100% for glioma (31). Intraoperative cytology smear may provide a qualitative intraoperative diagnosis in over 25% of cases where frozen sections yield a diagnosis of “equivocal brain tumor” (19). Notably, frozen sections are generally considered superior to cytological methods for intraoperative diagnosis. Frozen sections can present tissue structure characteristics more clearly, providing certain advantages in determining tumor type and boundaries (35). When diagnosis using frozen sections during stereotactic biopsy is difficult, cytological methods may serve as an alternative.

This study was associated with several limitations. First, the retrospective design may have led to information and selection biases. Second, the sample size from a single center limited the generalizability of the findings and statistical power. Third, intraoperative frozen section assessments are subjective and may vary between pathologists, potentially affecting outcomes.

## 5 Conclusion

This study shows the diagnostic yield of intraoperative frozen sections obtained through robot-assisted stereotactic biopsy of brain lesions is high. When initial results are negative, additional specimens should be obtained by adjusting either puncture depth or target location. Lesions that are difficult to distinguish morphologically on HE staining may be examined using intraoperative immunohistochemistry, although this will increase operative time. High grade glioma may be more prone to tentative or misdiagnosis due to heterogeneity of the lesion.

## Data Availability

The raw data supporting the conclusions of this article will be made available by the authors, without undue reservation.
